# Social structuring of the gut microbiome in communally roosting bats

**DOI:** 10.1371/journal.pone.0325710

**Published:** 2025-07-03

**Authors:** Eleonore Lebeuf-Taylor, Alexandria Cosby, Quinn Webber, Karl Cottenie

**Affiliations:** Department of Integrative Biology, College of Biological Sciences, University of Guelph, Guelph, Ontario, Canada; University of Reunion Island, RÉUNION

## Abstract

The gut microbiome is the community of microbes that inhabits the gastrointestinal tracts of animals. Laboratory findings have shown that the gut microbiome plays a crucial role in host metabolism, physiology, and immunity. This has led to speculation that selection acts on both host and microbiome—although identifying functionally essential coevolving microbes in wild animals remains challenging. A recent surge of studies in wild populations has identified phylogenetic, spatiotemporal, dietary, and social patterns in host-associated microbiomes. Here, we describe and assess the gut microbiomes of two sympatric bat species: big brown bats (*Eptesicus fuscus*) and little brown bats (*Myotis lucifugus*). Although these species share similar diets and environments throughout much of their North American ranges, we found they have distinct gut microbiomes. We find no evidence of a functional core microbiome among big brown bats and identify roost identity as a driver of microbiome composition, likely arising from social transmission among hosts through physical proximity. We conclude that both environmental and social factors drive microbiome composition in big brown bats and that repeated, extensive sampling is required to bring ecological reality to host-associated microbiome studies in wild populations.

## Introduction

The gut microbiome is a symbiotic community of microorganisms that inhabits the gastrointestinal tract of animals. It plays an important role in host physiology, including the development and function of host metabolism [1], immune system [2], and social behaviour [3]. However, the majority of microbiome findings stem from work in laboratory mice whose microbiomes are highly controlled; thus, the ecological reality of microbiome importance and function in wild populations is still under debate [4]. There is evidence for phylosymbiosis—the coevolution of host and microbiome—across disparate host taxa [5,6], suggesting that the microbiome, while dynamic, can coevolve with its host. To date, however, most microbiome studies in wild animal populations have focused on identifying the biotic and abiotic factors that drive variation in microbiome composition; diet and the local environment have emerged as common predictors across a broad range of host taxa [7,8].

Host sociality is also thought to be related to variation in microbiome composition. Experiments on laboratory mice have shown reciprocal effects: while the microbiome is essential for the development of normal social behaviour [3], social interactions can also shape the microbiome [9]. Social proximity may be beneficial to the host, as it increases the diversity of the microbial community and thus, it is thought, its resilience and functional redundancy [10–12]. Equally, from the perspective of host-associated microbial communities, social groups offer large pools of potential hosts [13] that offer new colonization opportunities. The interplay between these two factors has led to the suggestion that microbes that drive sociality are selected for in host taxa where sociality increases fitness [14] and the coining of the term ‘social microbiome’, which refers to the microbes acquired by hosts through social interactions [15]. While experimental evidence is still lacking, social structuring of the microbiome has been observed in wild systems: in mammals, for example, social networks can drive variation in microbiome composition [16–20]. However, the social microbiome is by no means a universal phenomenon [21,22] and sociality is only one of many factors that influence microbiome assembly.

Bats (order Chiroptera) form a highly diverse taxonomic group comprising over 1,400 species. Bats are among the most gregarious mammalian orders, with some species living in colonies exceeding a million individuals, which can have profound implications for viral and bacterial species richness [23,24]. Moreover, bats live and roost in a wide range of natural and anthropogenic structures [25], which has potential to influence pathogen dynamics [26]. Bats therefore offer an ideal framework to explore the relative contributions of phylogeny, environment, and social structure to wild animal microbiomes. Studies of bat gut microbiomes have revealed important differences from those of other animal taxa; there is limited evidence of phylosymbiosis—i.e., the coevolution of host and microbiome—in bats [27], contrary to what has been found in other mammalian orders [5,28]. The bat skin microbiome is shaped by uptake of environmental microbes [29], which may play a role in gut microbiome assembly via self- and allogrooming behaviours [21]. Diet has also been identified as a driver of bat gut microbiome composition: bat species that feed on aquatic resources have a distinct microbiome compared with insectivorous species [30,31]. This finding, however, is not generalizable, as studies have shown both presence [32] and absence [33,34] of microbiome similarity between dietary groups within Chiroptera. The main drivers of microbiome composition in bats remain, therefore, an area of active research.

The social structure of some bat species may also affect the composition of their microbiome. There is strong evidence for social convergence of the gut microbiome in vampire bats (*Desmodus rotundus*), wherein individuals who share blood meals display similar microbial profiles independent of kinship and shared environment [20]. In the colony-living and allogrooming Egyptian fruit bat (*Rousettus aegyptiacus*), fur microbiomes converge at the colony level as do, to a lesser degree, their gut microbiomes [21]. In theory, the benefits of group-living in bats—for instance, as a way to conserve body heat [35,36]—could extend to microbial transmission among conspecifics. Colonies may therefore act as pools for the horizontal transmission of microbes between conspecifics; this may help maintain essential symbionts and a degree of microbial richness, which is thought to be important for resilience to perturbations [37,38].

Big brown bats (*Eptesicus fuscus*) and little brown bats (*Myotis lucifugus*) are widespread throughout temperate North America. While they are deceptively alike in their common names, the two species diverged ~33 million years ago [39]. Nevertheless, both are insectivorous, hibernate throughout the winter, and have similar ecological niches and life histories*.* Big brown bats and little brown bats form maternity colonies in spring and early summer, where they give birth to and rear their pups. Maternity colonies tend to be fission-fusion societies: subgroups merge and split through time, and individual bats may occupy many different roosting sites [35,40]. Given their ecological and behavioural similarities, it might be expected that big brown bats and little brown bats have similar gut microbiomes. Alternatively, their relatively long ancestral divergence may have led their microbiomes to diverge. Their group-living social structure may further shape the composition of their microbiomes. While previous work in big brown bats and little brown bats has investigated their skin microbiome in relation to the fungal pathogen that causes white-nose syndrome [29], their gut microbiomes, and associated sources of variation, have not yet been described.

**Fig 1:**
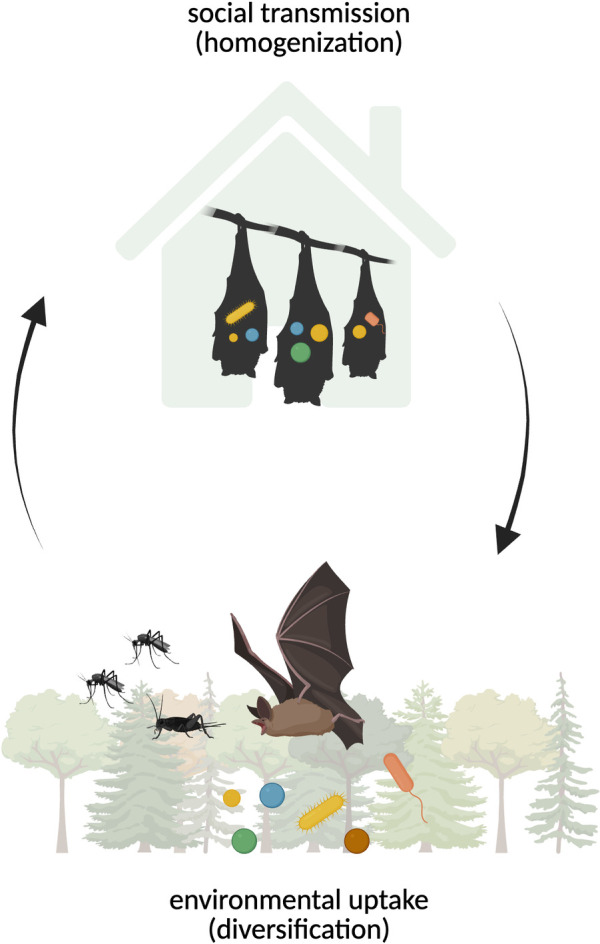
Graphical hypothesis of gut microbiome assembly in big brown bats and little brown bats. Horizontal transmission, via environmental sampling and roost-level clustering, may shape microbiome community assembly in big brown bats and little brown bats. Created in BioRender. Lebeuf-Taylor, E. (2025) https://BioRender.com/eo0eybs.

Our primary aim is to describe and identify the drivers of gut microbiome variation between and among big brown bats and little brown bats, with a particular focus on the effects of group living. We posit that two main processes drive microbiome variation, both of which occur through horizontal transmission (Fig 1). The first process diversifies the microbiome via environmental uptake: while foraging, individual bats sample the wider regional microbial species pool, taking up microbes through environmental contact and diet. The second process homogenizes the gut microbiome at the colony level: during daytime roosting, individuals likely exchange microbes through a defecation-grooming route akin to that proposed in Egyptian fruit bats [21]. Additionally, it stands to reason that the larger the colony, the wider the sampling of the regional microbial species pool; thus, in the presence of horizontal transmission between roost mates, we could expect to see higher microbial community richness (alpha diversity) in bats living in larger colonies.

The diversifying process may be stronger than the roost-level homogenization. In this scenario, temporal and spatial factors are the main drivers of differences in microbiome composition. Seasonality in the biotic environment and/or host energetic demands (including reproductive status) could affect which microbes are acquired from the environment and whether they are maintained in the host. Heterogeneous distribution of resources may also lead to distinct microbiomes as individuals differentially sample the regional microbial species pool without transmitting microbes between themselves: in this case, individual hosts are metaphorical islands for their bacterial community.

Functionally important microbes may be conserved across individuals. Microbial taxa that are essential to host function may be highly prevalent (that is, found in most samples) and relatively abundant despite the ongoing processes of environmental uptake and social transmission. Thus, conserved bacterial taxa could constitute a common core microbiome. Given the evidence for diet-related similarities in some bat gut microbiomes [27,30], we expected the core microbiomes of big brown bats and little brown bats, as geographically overlapping insectivorous bat species, to overlap.

## Methods

### Sample collection

**Fig 2:**
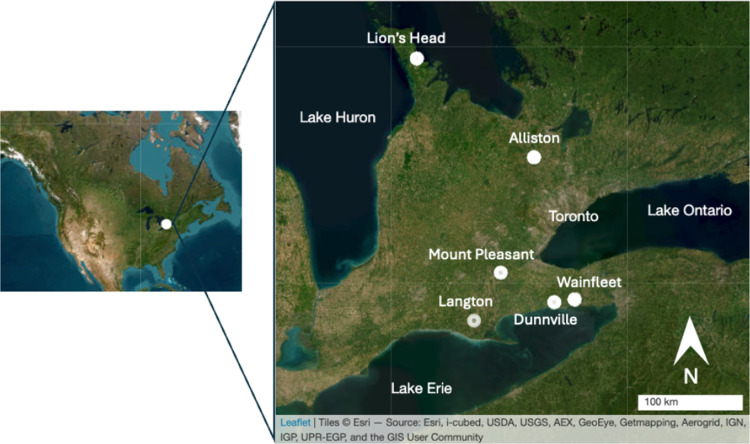
Locations of the six maternity colonies sampled across southern Ontario, Canada. Map data copyrighted OpenStreetMap contributors and available from www.openstreetmap.org [41].

Bats were caught from six maternity roosts (Fig 2) between 5 June 2023 and 15 August 2023 using funnel traps and harp traps placed at the entrance of each roost. Traps were set up 30 minutes to an hour before sunset and left open for up to four hours, weather permitting. Individuals were removed from traps or nets within 10 minutes of capture and faecal samples were opportunistically collected from spontaneous defecation in a clean terrycloth bag while bats were awaiting processing or while bats were in hand. Samples were collected and stored on ice for the duration of the field trip (maximum three days), then placed in a −20°C freezer upon return from the field. All bat captures and handling were approved through University of Guelph Animal User Protocol AUP-4958. Access to field sites was granted through a Wildlife Scientific Collectors Authorization permit from the Ontario Ministry of Natural Resources and Forestry.

All colonies were single-species except the Langton colony, which included both big and little brown bats. Roost sites varied in size and included buildings and bat boxes. The Wainfleet and Lion’s Head sites were large big brown bat and little brown bat (respectively) maternity colonies in garages (N ~ 150 bats total in each, n = 20 and n = 13 fecal samples collected for this study respectively). The Dunnville site was a smaller big brown bat maternity colony in a garage (N ~ 40 bats total, n = 8 captured). The Langton site was a large mixed-species colony (big brown bats and little brown bats) in a barn (N > 100 bats total, n = 2 big brown bats captured). The Alliston and Mount Pleasant sites were small big brown bat maternity colonies in bat boxes (N ~ 40 bats each, n = 17 captured and n = 6 captured, respectively). The big brown bat samples were collected from 40 adults and 13 juveniles, whereas all little brown bats sampled were adults.

### DNA extractions, sequencing, and quality control

DNA extractions and sequencing were performed at the McMaster Genomics Facility following a modified version of the protocol described in Stearns et al., 2015 [42]. Briefly, 2x300bp paired-end reads were generated from amplification of the V4 region of the 16S rRNA gene using 515F and 806R primers. A negative control was used. Sequencing was performed on the Illumina MiSeq platform as described in [43] at the McMaster Metagenomics Facility (Hamilton, ON, Canada). Cutadapt was used to filter and trim adapter sequences and primers from the raw reads, with a minimum quality score of 30 and a minimum read length of 100 bp [44]. Sequence variants were filtered and trimmed based on the quality of the reads, error rates were learned, and sequence variants were called using DADA2. Bimeras were removed and taxonomy was assigned using the RDP classifier against the SILVA database version 1.3.8. Two ASVs were detected in the negative control with counts of 7 and 8, which were subtracted from the final ASV table.

### Statistical analyses

All analyses were conducted in R (version 4.3.0 [45]) using the packages phyloseq [46] and vegan [47]; dominant taxa were identified using the R package fantaxtic [48], core microbiomes using the R package microbiome [49], and differential abundances using the R package ALDEx2 [50]. We calculated alpha diversity using observed ASVs and used a linear mixed-effect model to assess the effect of colony size with colony identity as a random effect.

We visualized beta diversity and tested predictor variables via distance-based redundancy analyses (db-RDA) using the capscale function from the vegan package on robust Aitchison distances [51]. The robust Aitchison calculation, with its centre log ratio (clr) transformation, has been specifically developed for microbial community data, which is sparse and contains large amounts of rare taxa [51]. We used a principal coordinates of neighbourhood matrices (PCNM) analysis to test for several spatial patterns at different scales [52]. We used the ordiR2step function from vegan to perform model selection. We used ALDEx2 on clr-transformed values to identify ASVs that were differentially abundant between big brown bats and little brown bats, with a Benjamini-Hochberg correction for multiple comparisons.

We tested for evidence of a core microbiome using a combination of relative abundance and prevalence across samples, with a minimum threshold prevalence of 75%. Due to the sparse nature of microbiome data, low sample size, and large variation among samples, we compared different thresholds of prevalence and relative abundance rather than relying solely on high prevalence and high abundance. Since it is unclear whether relative abundance is directly correlated to functional importance [53], a sliding-scale approach allows for the use of various thresholds without making assumptions about relative importance.

## Results

We collected 66 samples from six roosts across southern Ontario, of which 53 faecal samples were from big brown bats across five roosts (mean number of samples per roost: 9) and 13 from little brown bats from one roost (Fig 2). After quality control and filtering, a total of 4,316,925 16S rRNA amplicon libraries were generated with a mean of 65,407 reads per sample (±35,057 standard deviation). Among big brown bats, 1978 amplicon sequence variants (ASVs) were identified, while 254 ASVs were detected in little brown bat samples.

**Fig 3:**
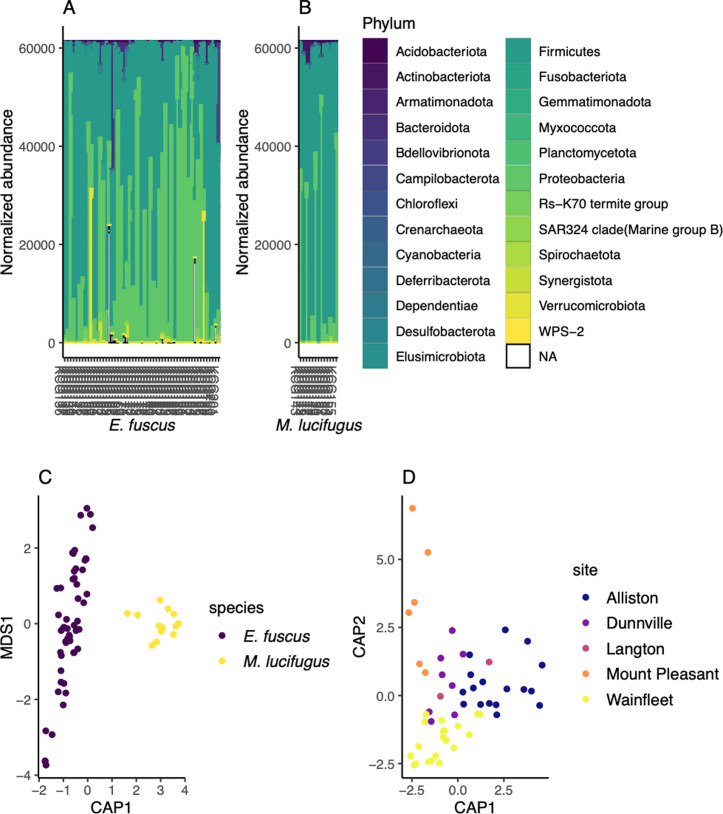
Composition and drivers of variation in the gut microbiomes of big brown bats and little brown bats. Phylum-level characterization of taxa in samples from **(A)** big brown bats and **(B)** little brown bats*.* **(C)** Host species identity significantly explained 6% of variation (db-RDA, *F* = 5.13, *p* = 0.001); **(D)** Maternity colony site within the big brown bat population explained 4% of variation (db-RDA, *F* = 1.49, *p* = 0.002).

The microbiomes of both species were dominated by the phyla Firmicutes and Proteobacteria, with mean relative abundances of 56% and 37% respectively in big brown bats and 70% and 27% in little brown bats (Fig 3). In both species, the third most abundant phylum (Verrumicrobiota in big brown bats and Actinobacteriota in little brown bats) lagged far behind, representing only 2% of ASVs in both host species. Despite higher-order similarities in their microbiomes, the two bat species exhibited stark differences at the genus level: in big brown bats, the top three genera were *Enterococcus* (25%), *Klebsiella* (10%), and *Lactococcus* (10%), whereas in little brown bats these were *Clostridium sensu stricto 1* (22%), *Plesiomonas* (20%), and *Paeniclostridium* (10%). Altogether, 208 ASVs were shared between big brown bats and little brown bats.

**Fig 4:**
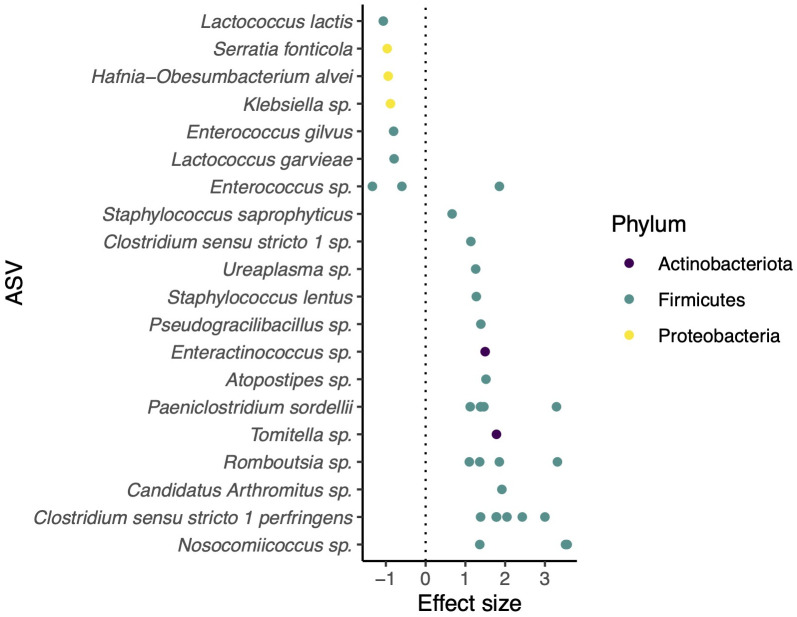
ASVs driving community composition differences between big brown bats and little brown bats. Negative effect sizes indicate ASVs enriched in big brown bats and positive effect sizes indicate ASVs enriched in little brown bats.

There was a significant difference in microbiome composition between big brown bats and little brown bats (db-RDA, *F* = 5.13, *p* = 0.001; Fig 3C). Specifically, we identified 44 differentially abundant ASVs (Fig 4). Of these, nine ASVs were found to be significantly more abundant in big brown bats, while little brown bat samples had 35 differentially abundant ASVs, the majority of which belonged to the phylum Firmicutes. Among *Enterococcus* ASVs identified to the genus level only, two were more abundant in big brown bats and one in little brown bats.

**Fig 5:**
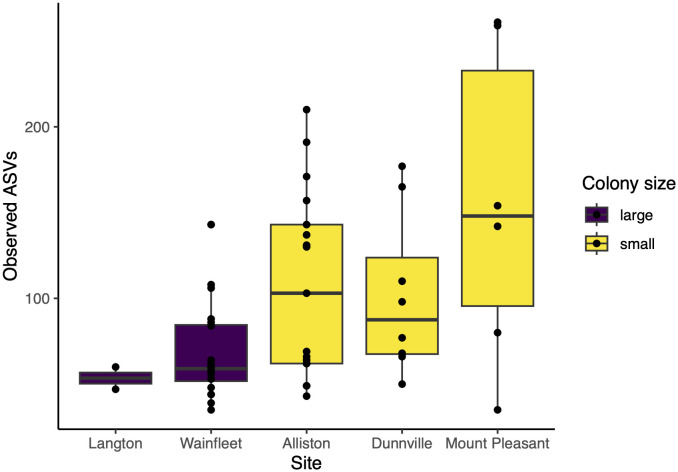
Alpha diversity of the gut microbiome of big brown bats across five maternity roosts. Alpha diversity did not vary significantly by colony size after accounting for colony identity (GLMM with colony identity as random effect, β = 53.47, standard error (SE) = 21.43, *z* = 2.49, *p* = 0.17).

In light of the significant difference in the gut microbial communities of big and little brown bats—and given the small sample size of the latter—we restricted subsequent analyses of predictor variables and core taxa to big brown bats only. Contrary to our prediction, alpha diversity measured in observed ASVs appeared higher in smaller colonies (n = 3) than in larger ones (n = 2). However, colony size was not significant once we accounted for site identity as a random effect (generalized linear mixed model, β = 53.47, standard error (SE) = 21.43, *z* = 2.49, *p* = 0.17; Fig 5).

Microbiome composition in big brown bats differed significantly according to colony identity (db-RDA, *F* = 1.49, *p* = 0.001, based on 999 permutations, Fig 3D), but not spatial factors (db-RDA on PCNM-generated distances, *F* = 1.77, *p* = 0.06, based on 999 permutations). To account for the possibility that roost-level convergence was driven by vertical transmission and/or mothers grooming their offspring, we repeated this test with the exclusion of juveniles and found that the difference was maintained (db-RDA, *F* = 1.46, *p* = 0.02, based on 999 permutations). We also found that, overall, time was a significant predictor of microbiome composition (db-RDA on Julian date, *F* = 1.73, *p* = 0.003, based on 999 permutations), although it is important to note that some sites were only measured once during the sampling period. The final model included both colony identity and seasonal variation, which together explained 4.3% of the total variance in community composition (adjusted R^2^ = 0.043, *p* = 0.038). Site was a stronger predictor than Julian date, explaining some 3.6% of the total variance, whereas Julian date explained an additional 0.7%.

**Fig 6:**
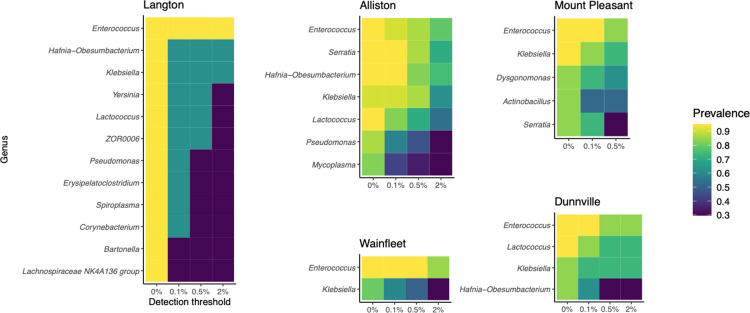
Bacterial genera identified as core in big brown bat roosts with a minimum prevalence of 75% of samples. Detection thresholds indicate minimum relative abundances; Langton n = 2; Mount Pleasant n = 6; Dunnville n = 8; Alliston n = 17; Wainfleet n = 20.

We performed core microbiome analyses for each colony separately given the significant difference between sites. There was very little overlap in core genera between colonies and, overall, very low relative abundances of core genera (Fig 6). Only *Enterococcus* was identified as part of the core microbiome in all five sites. *Lactococcus*, *Hafnia-Obesumbacterium*, and *Klebsiella* were each found in three roosts—though not always co-occurring—at varying prevalences and relative abundances. To identify the bacterial taxa driving roost-level differences, we performed a differential abundance analysis on the two colonies with sample sizes large enough to justify such a test (Alliston and Wainfleet), which identified *Serratia fonticola* as the single differentially abundant ASV.

## Discussion

Our objective was to describe, compare, and identify the drivers of variation in the gut microbiomes of big brown bats and little brown bats. We found that despite substantial interindividual variation, microbial communities differed significantly by host species and that both social and temporal—but not spatial—factors explained variation in community composition (Fig 4). We did not identify a core group of bacterial taxa that were conserved across maternity roosts in big brown bats; rather, taxa identified as core in individual colonies were present in relatively low prevalences and abundances (Fig 6). Taken together, our results suggest that both environmental and group-level processes govern microbiome assembly in these bat species.

Big brown bats and little brown bats displayed significantly different gut microbiome profiles (Fig 3) and differential abundance analysis revealed fundamental differences between the host species (Fig 4). Indeed, several of the ASVs that were significantly enriched in big brown bat gut microbiomes were also identified in the core microbiomes of many big brown bat colonies—indicating very limited interspecific overlap in microbiome profiles. Furthermore, although the number of ASVs detected in little brown bats was close to one-tenth of those found in big brown bat samples, a higher proportion of ASVs were significantly enriched in little brown bats. This suggests that little brown bats harbour several taxa that are very rare, or completely absent, in the big brown bat gut microbiome—despite the latter’s much larger sample size (Fig 4). We note that among three *Enterococcus* ASVs unidentified at the species level, two were enriched in big brown bats and one in little brown bats, which underscores the value of identifying microbial taxa with ASVs rather than collapsing to higher taxonomical orders.

In both big brown bats and little brown bats, the gut microbial community was dominated by the phyla Firmicutes and Proteobacteria, similar to what has been observed in both frugivorous and insectivorous bats [21,33,54]. This higher taxonomic order description is not particularly informative, however, as Firmicutes and Proteobacteria are large, highly diverse taxa that include both commensals and pathogens. At the genus level, the top genera in big brown bats, *Enterococcus* and *Lactococcus*, were also found to dominate the gut microbiome of other insectivorous bat species [55]; both taxa play a vital role in digestion in mammals [56] and are known to ferment carbohydrates [57]. The dominant genus in little brown bats, *Clostridium sensu stricto 1*, is also a fermenting taxon that has been detected in high abundances in omnivorous bats [58]. Notably, *Clostridium sensu stricto 1* is a known enteric pathogen in other host taxa [59,60], highlighting the host-specific roles of microbial taxa.

We did not find evidence that big brown bats and little brown bats share a common overlapping core microbiome, although we only sampled a single little brown bat roost. While roost site confounds species identity, we nonetheless detected a clear species-level clustering (Fig 3C). If the gut microbiomes of big brown bats and little brown bats are at least partially colonized by ingested inocula, dietary differences between the two species could explain some degree of dissimilarity; while both species are insectivorous, big brown bats prey heavily on Coleoptera [61] and little brown bats mainly feed on Ephemeroptera [62]. Increased sampling of little brown bats is necessary to better understand how, or indeed if, their microbiome differs from that of big brown bats and to explore whether they also exhibit colony-level clustering.

Despite the differences in community composition between big brown bat roosts, we expected that functionally important taxa may be retained as part of a common core. This was not the case, as core analyses revealed large differences between roosts (Fig 6). However, it is important to note that functional redundancy among microbes, which can be partly due to horizontal gene transfer (i.e., exchange of genetic material between unrelated bacteria) [12,63], can result in microbial communities that are taxonomically different yet functionally similar. Previous work in multiple bat species has detected a convergence of metagenomic function, but not community composition, according to diet [64,65]. Any such functional redundancy would not be identifiable in our study, which consists of a taxonomic inventory of the microbial community. Metagenomic characterization of the big brown bat gut microbiome is a promising avenue for future work in this population.

The only microbial genus found in all five roosts was *Enterococcus*, which is a large and highly diverse clade that includes common gut symbionts of humans, wild vertebrates, and insects, suggesting a long history of coevolution with animal hosts [56,66,67]. *Enterococcus* is particularly widespread in bats and vertebrates with varied diets [56], though whether bacteria from this genus fulfill a metabolic role in the bat gut has yet to be determined. Indeed, the presence of *Enterococcus* in all sites is more revealing of its pervasiveness in animal microbiomes rather than an indication that it fulfills any particular functional role. That said, antibiotic-resistant strains of *Enterococcus* have been found in bat faeces in Europe [68], which warrants closer monitoring of the big brown bat microbiome for potentially pathogenic strains.

The unexpected lack of overlap in core taxa between colonies (Fig 6) was maintained even when focusing only on the sites with the most samples, which were sampled repeatedly throughout the summer. This finding raises the question of whether it is meaningful to describe a core microbiome for big brown bats at all: if some symbionts were truly core—in the sense that they are essential to the host—we may expect to find them in all sites. To our surprise, further analysis revealed that the only differentially abundant taxon between Alliston and Wainfleet was *S. fonticola*, which was also identified as core in Dunnville and Mount Pleasant. If bacterial taxa are indeed taken up from the environment and homogenized at roosts, it is possible that longitudinal sampling might reveal change in differentially abundant taxa every season corresponding to variation in the regional microbial pool and a hibernation-related microbiome alteration. Altogether, these observations suggest a large degree of variation in microbiomes and high uncertainty as to the validity of ascribing a functionally meaningful ‘core’ designation to any particular taxon.

The absence of evidence for a common core between both species and across colonies suggests that these bats have not evolved a tight association with any particular symbionts. The fact that these species are generalist feeders may be the cause: the diversifying effect of variation in food sources between individuals, across time and space, could mean that there is variable uptake from environmental sources. Furthermore, the absence of consistent resources in the gut environment—which would occur in a specialist diet—would result in weaker selective pressure for any particular microbial taxa in the gut. Without consistent inoculation and selective pressure favouring particular bacterial taxa, the development of a tight evolutionary link between microbes and host is less likely.

Our findings further underscore the importance of sampling widely across groups in populations with a social structure. Typically, microbiome studies in wild systems—compared with laboratory studies—are characterized by relatively low sample sizes and uneven sampling across temporal and spatial scales. Although the concept of a core microbiome is an appealing one, this study highlights the importance of undertaking a core microbiome analysis that is informed by known sources of variation. While a core microbiome for big brown bats may exist, identifying it may only be possible with greater sample sizes across a wide variety of predictors.

Contrary to our expectations, we found that smaller roosts did not have lower alpha diversity than large roosts in big brown bats. While this observation may be biased by small sample sizes (there were three small colonies and two large colonies), it may nevertheless reflect greater horizontal transmission in smaller roosts. Microbes may be horizontally transmitted when faeces are splattered inside the roost and on other individuals [21]. In small colonies, which roost in smaller, more enclosed spaces like bat boxes, there may be greater social contact among hosts and thus increased transmission wherein individuals inoculate each other. Two of our small colonies do indeed roost in bat boxes. Larger colonies, on the other hand, may spread out more in open spaces like garages, with less opportunity for transmission. In wild mammal populations more generally, no consistent link between group size and alpha diversity has been established: in baboons, larger group sizes have been associated with increased alpha diversity [69], but this is not the case in red-bellied lemurs [70]. The ecological relevance of either finding is unknown: despite the role that richness plays in resilience in macroecological communities, whether higher alpha diversity in the microbiome is associated with improved outcomes for the host has yet to be investigated.

Our analysis of the big brown bat microbiome revealed colony-level clustering (Fig 3) consistent with work in other bat species. This finding is not entirely unexpected, given that physical contact facilitates horizontal transmission between external microbiomes [21], as does fluid exchange for internal microbiomes [20,71]. However, our results show that colony-level similarities can also develop in the absence of fluid exchange. The physical proximity that allows for the homogenization of the fur and skin microbiome in fruit bats [21] may also drive colony-level gut microbiome similarities in big brown bats via the presence of faeces in the roost and inoculation through self-grooming and physical contact. A defecation-grooming route entails that gut microbes must survive outside the gastrointestinal tract, which favours facultative anaerobes (although some anaerobes can survive in the presence of oxygen for several hours [72]). Indeed, several of the more prevalent taxa identified (e.g., *Enterococcus*, *Hafnia*, *Klebsiella*, *Lactococcus*, *Serratia*) are facultative anaerobes [57,73]. Ultimately, the colony-level convergence suggests that indirect horizontal transmission of gut microbes may be an underappreciated factor contributing to group-level homogenization in bat gut microbiomes.

It remains nonetheless possible that diet, rather than social transmission, drives intraspecific roost-level differences. We identified a temporal pattern to microbiome profiles, which may reflect the transition from hibernation to the active season as well as seasonal changes in resource availability. Studies in hibernating mammals, including bats, have indeed identified differences between hibernation and the active season, where alpha diversity is consistently reduced during hibernation [74–76]. Furthermore, roost-level clustering may be due to distinctive, localized food sources that may act either as probiotics (a dietary source that fosters the growth of particular bacteria taxa) or inocula (i.e., wherein the bat gut is colonized by the symbionts of the bats’ prey). More generally, understanding the contribution of hibernation and seasonal dietary variation to the bat gut microbiome requires much deeper investigation, with a particular focus on both longitudinal sampling and comparing bats’ microbiome to that of their prey (as potential inocula) and identifying the metabolic role, if any, of their gut microbes through metagenomics.

Overall, this study raises several further questions that will only be possible to address with wider sampling. Our explanatory variables—roost identity and time—explained some 4% of variation among microbiomes. Additional predictor variables can only be tested once sufficient sample sizes have been attained to prevent multicollinearity. For instance, roost types and colony sizes may be impossible to distinguish if too few colonies are sampled, as is the case here. Future work could test interactions between roost type and colony size, as well as the prediction that roost type affects colony-level microbiome convergence, since boxes generally allow tighter quarters than buildings and thus potentially greater colony-level homogenization. Repeated sampling at the same locations over the course of a season will also help untangle the relative contribution of temporal and spatial factors. Finally, any future work on gut microbiomes of bats will be greatly enhanced by detailed assessments of their diet, to understand both the contribution of environmental inocula and any diet-specific microbial symbioses.

## Conclusion

Our study combines inter- and intraspecific analyses of the gut microbiome of two bat species with similar ecological niches. We show that, despite sharing a similar diet and environment, big brown bats and little brown bats have distinct microbiomes. Among big brown bats, social structuring of the population into maternity roosts is an important driver of microbiome convergence, despite the absence of fluid exchange. We also find a temporal component to microbiome composition, which warrants further investigation into the seasonal patterns of microbiome profiles via repeated sampling of individual roosts. Despite strong evidence from laboratory studies that the gut microbiome is essential for several aspects of host function, we see no evidence of this phenomenon in big brown bats, as we did not identify core bacterial taxa found at high prevalences and relative abundances across roosts.

Untangling the causal patterns of social structure and the microbiome in big brown bats represents a promising avenue for future work. This could be achieved, for example, with transplant experiments, whereby bats from different roosts are swapped and their microbiomes tracked to see whether a colony-level convergence emerges. Incorporating social interaction data will also be crucial to understanding how, if at all, opportunities for horizontal transmission arise both inside and outside the roost. Finally, linking fitness proxies to the microbiome will yield important information on the role of the microbiome in adaptation and the applicability of the holobiont concept to this host species.
